# Supplementary material for: Influence of the Composition of the Hybrid Filler on the Atomic Oxygen Erosion Resistance of Polyimide Nanocomposites
Source: Materials (Basel). 2020 Jul 18;13(14):3204. doi: 10.3390/ma13143204 (PMC7411669; doi:10.3390/ma13143204)
Supplement: Supplementary file 1 [file materials-13-03204-s001.pdf]

materials

MDPI

Article
Supplementary Materials

Influence of the Composition of the Hybrid Filler on the Atomic Oxygen Erosion Resistance of Polyimide Nanocomposites

Olga Serenko [1,*], Ulyana Andropova [1,2], Nadezhda Tebeneva [2], Mihail Buzin [1], Egor Afanasyev [1], Aleksander Tarasenkov [2], Sergey Bukalov [1], Larisa Leites [1], Rinat Aysin [1], Lev Novikov [3], Vladimir Chernik [3], Ekaterina Voronina [3] and Aziz Muzafarov [1]

[1] A.N. Nesmeyanov Institute of Organoelement Compounds of Russian Academy of Sciences, 28 Vavilova St., GSP-1, V-334, Moscow 119991, Russia; hrh_uly@mail.ru (U.A.); buzin@ineos.ac.ru (M.B.); nambrot@yandex.ru (E.A.); buklei@ineos.ac.ru (S.B.); raman@ineos.ac.ru (L.L.); aysin@ineos.ac.ru (R.A.); aziz@ineos.ac.ru (A.M.)
[2] N.S. Enikolopov Institute of Synthetic Polymeric Materials of Russian Academy of Sciences, 70 Profsoyuznaya St., Moscow 117393, Russia; tebeneva@mail.ru (N.T.); antarr@bk.ru (A.T.)
[3] D.V. Skobeltsyn Institute of Nuclear Physics, Lomonosov Moscow State University, 119991 Moscow, Russia; novikov@sinp.msu.ru (L.N.); vlachernik@yandex.ru (V.C.); voroninaen@nsrd.sinp.msu.ru (E.V.)
* Correspondence: oserenko@yandex.ru

Received: 26 June 2020; Accepted: 16 July 2020; Published: date

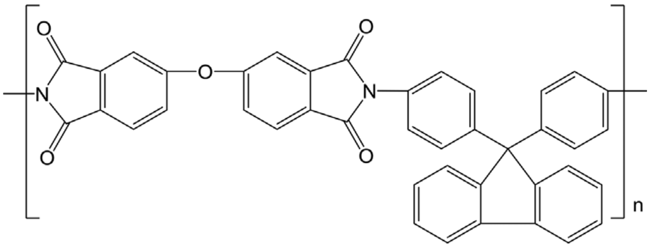

Figure S1. Chemical structure of polyimide. Polymer with M_w=10.7* 10^5 was synthesized by one-step high-temperature polycondensation of the relevant monomers in m-cresol. A reduced viscosity of 0.97 dL/g was measured for the polymer solution in N-methyl-2-pyrrolidone at 25 °C.

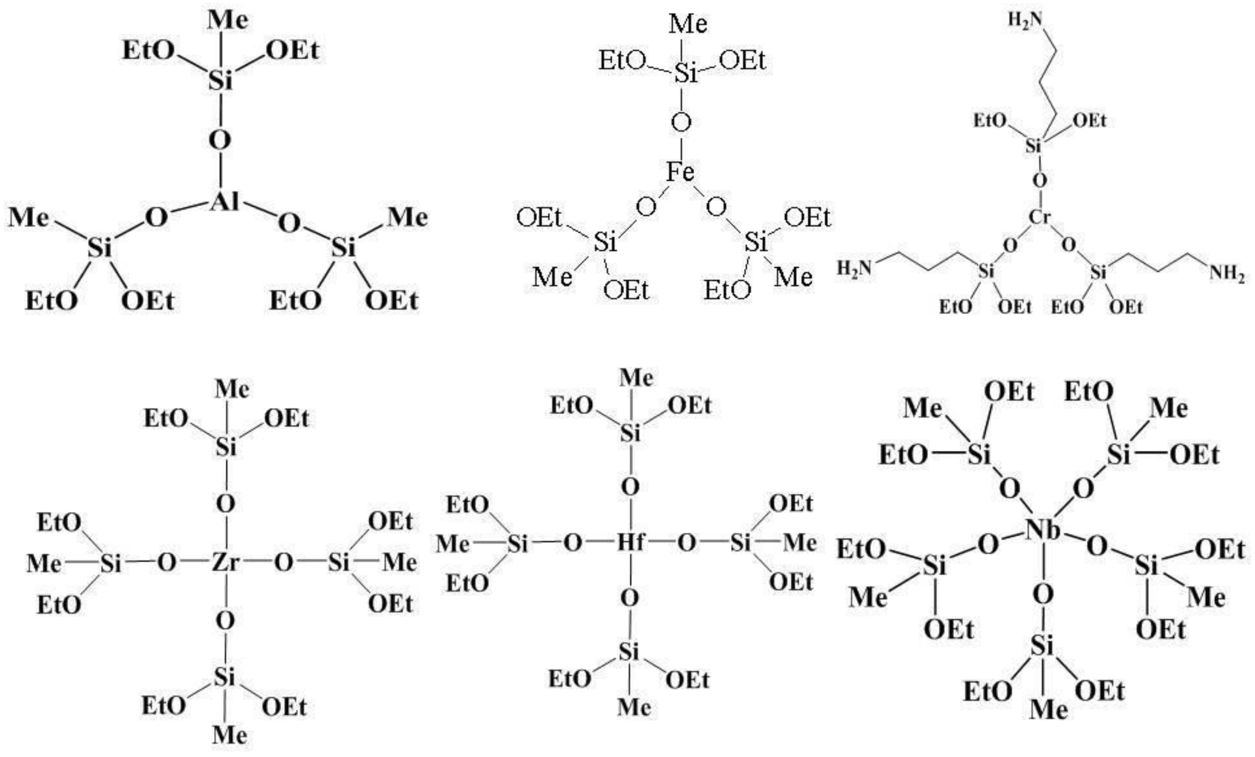

Figure S2. Structural formulas of precursors.

Table S1. Compositions of films based on PI *.

Sample	Precursor Concentration [wt.%]	Precursor Solution Weight [g]	Filler Content	
			[wt.%]	[mmol/g]**
PI-Al-	3	0.062	1.6	0.065
MDES	14	0.323	8.0	0.341
PI-Fe-	3	0.063	1.7	0.06
MDES	14	0.329	8.1	0.313
PI-Cr-	3	0.073	2.3	0.066
MDES	14	0.384	10.7	0.346
PI-Zr-	3	0.063	1.8	0.046
MDES	14	0.324	10.0	0.285
PI-Hf-	3	0.060	1.8	0.039
MDES	14	0.325	10.7	0.249
PI-Nb-	3	0.067	1.8	0.040
MDES	14	0.352	8.3	0.210

*The 5 wt. % precursor solution in chloroform was added to 0.10 g PI solution in 4 ml of chloroform with stirring in an argon stream. ** mmol of filler per gram of PI.

Preparation of filled films

A precursor solution in chloroform was added to 0.10 g PI solution in 4 ml of chloroform with stirring in an argon stream. The detailed formulations are listed in Table S1. The precursor concentration was 3 and 14 wt%. The resulting solution was poured into a teflon form and dried at room temperature for 3 days. Afterwards, the polymer film was heat treated in a drying oven with a gradual increase in temperature from 50 to 200 °C for 6 hours.

The thickness of the obtained films was 110-120 μm. The filler contents, according to calculations carried out under the assumption of 100% conversion of the ethoxy groups, are presented in Table S1.

For analyses of filled films, the polymer film and the film of 100% precursor, obtained under identical conditions, served as samples for comparison.

AO Beam Exposures

The facility consists of an evacuated vessel in which a plasma accelerator is placed. The vessel has a specimen holder and beam diagnostic equipment. Using vacuum pumping by cryogenic pumps with a rate of 5 m^3/s, the vessel maintains a pressure of (0.5-2)×10^{-2} Pa with a plasma-supporting gas-oxygen requirements of 0.5 L Pa/s. The beam components are atoms, molecules and oxygen ions with a predominance of atomic ions. The power flux density absorbed by the specimen was 15 mW/cm^2, which approximately corresponds to the heating of the specimen by solar radiation in space.

Film samples 20×20 mm in size were used. The specimens were degassed beforehand and held for 24 hours at a temperature of 20 °C in a vacuum of 10^{-4} Pa.

Specimens were irradiated by an oxygen plasma beam from a plasma accelerator, simulating low-earth orbit conditions. To ensure the same exposure, the analysed specimens and the reference specimen were mounted on a rotating disk, placed normally to the plasma flow. The specimens' masses were measured outside the evacuated vessel on an analytical microbalance HR-202i (AND, Japan) with a scale multiplier of 10^{-5} g, before and after each irradiation cycle with plasma flow.

In the experiment, the effective fluence method was used to determine the intensity of AO exposure [1]. The equivalent AO fluence, F (O atoms cm^{-2}), was determined by the change in weight of a reference sample (Kapton H polyimide film, DuPont) with an erosion yield of $E_K = 3 \times 10^{-24}$ cm^3/ atom:

$$F = \frac{\Delta m_K}{S \rho_K E_K} \quad (1)$$

where Δ m_K is the reference sample weight loss (g) during AO exposure, S is the exposed surface area (cm^2) and ρ_K is the density of the reference sample (1.42 g/cm^3). The effective AK flux density in polyimide equivalents was (3-4)×10^{16} atom/cm^2 s.

The erosion yield coefficient (E_y, cm^3 $atom^{-1}$) of research samples is defined as the volume loss caused by one AO attack, calculated by Eq. (2)

$$E_y = \frac{\Delta m}{S \rho\, F} \quad (2)$$

where Δm is the sample weight loss (g) during AO exposure, S is the exposed surface area (cm^2), and ρ is the density of sample (1.38 g/cm^3).

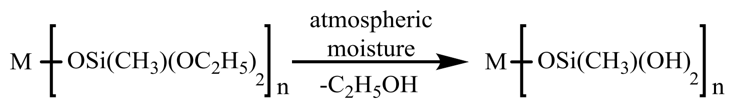

(a)

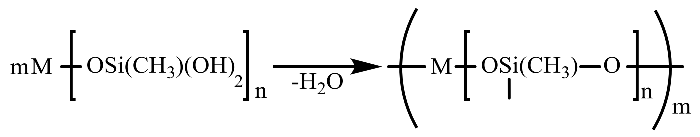

(b)

Figure S3. Assumed scheme of metalloalkoxysiloxane hydrolysis (a) and condensation (b), where M= Al, Fe, Zr, Hf, Nb and n=3-5.

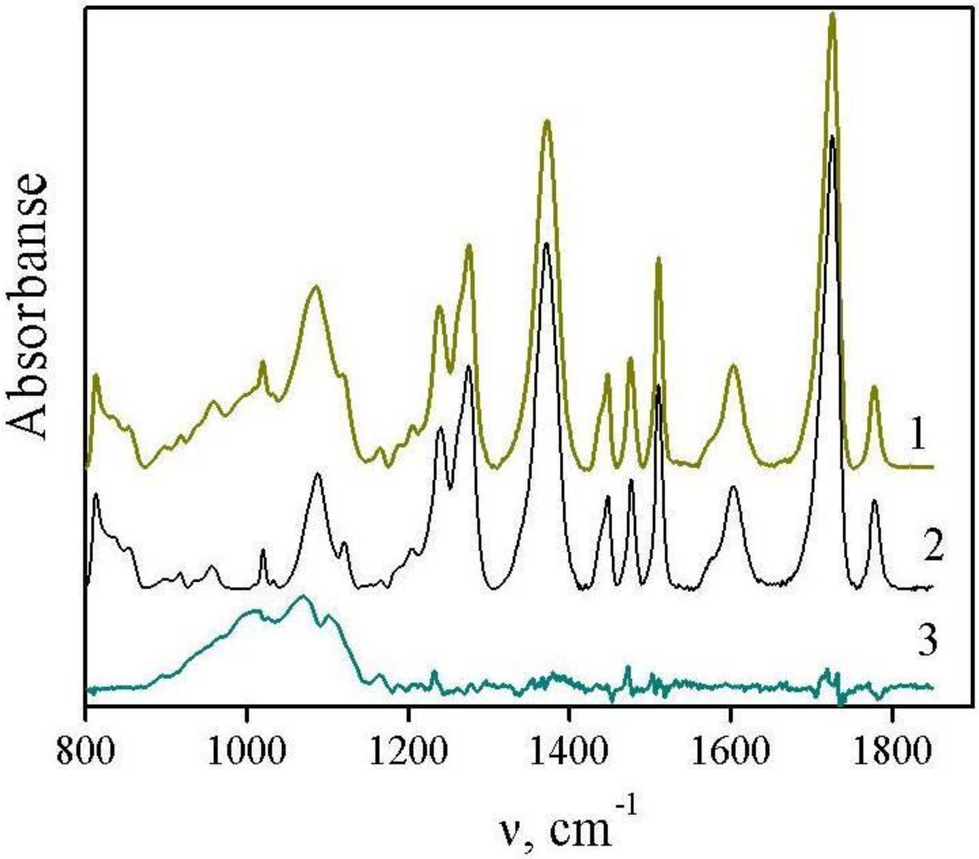

Figure S4. IR spectra of the films (on KBr plates) in the region 800-1800 cm^{-1}: the PI – 14 wt.% Cr-MDES (1), the PI (2) and the difference spectrum (3), proving the presence of Cr-MDES in the composite (a broad band 900-1150 cm^{-1} in the difference spectrum).

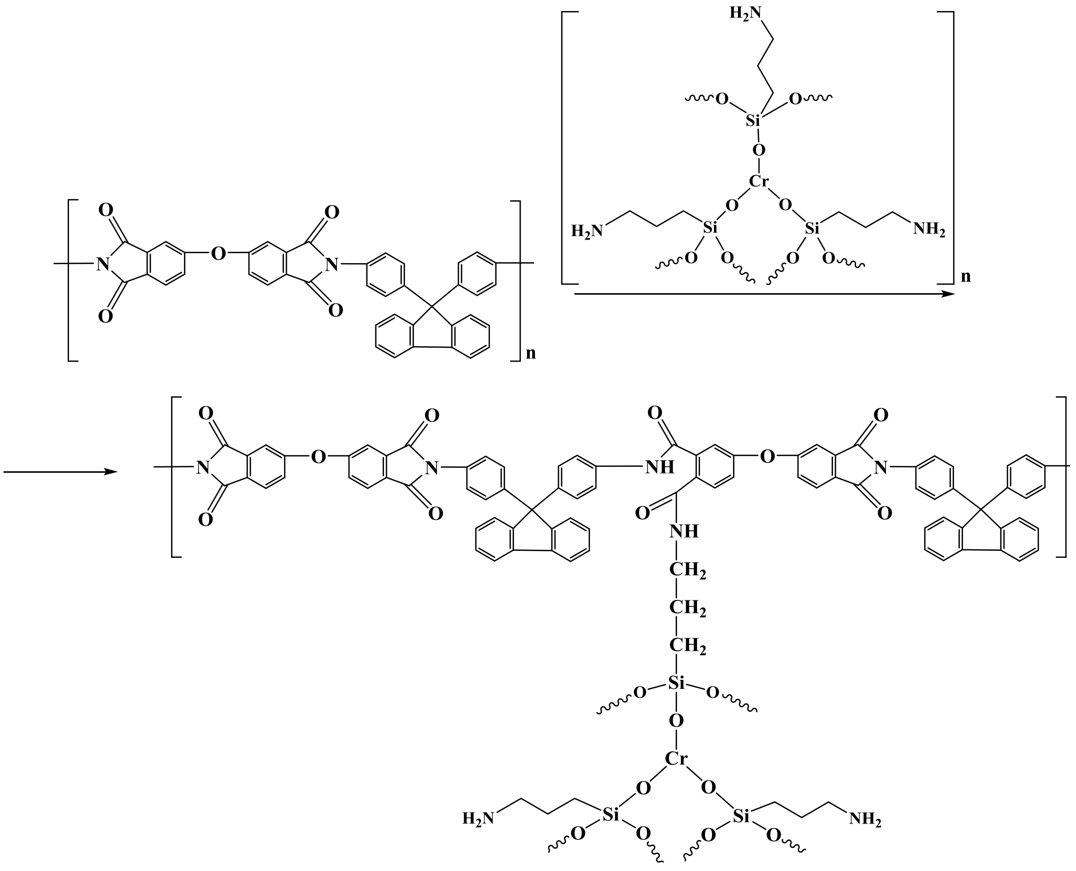

Figure S5. The proposed scheme of the reaction the amine group of the precursor with the imide group of the matrix polymer.

Reference

1. ASTM E. 2089-00. Standard practices for ground laboratory atomic oxygen interaction evaluation of materials for space applications, Annual book of ASTM standards, 2000.

© 2020 by the authors. Submitted for possible open access publication under the terms and conditions of the Creative Commons Attribution (CC BY) license (http://creativecommons.org/licenses/by/4.0/).
